# Recommendation for incorporation of a different lymph node scoring system in future AJCC N category for oral cancer

**DOI:** 10.1038/s41598-017-06452-0

**Published:** 2017-10-26

**Authors:** Ching-Chih Lee, Yu-Chieh Su, Shih-Kai Hung, Po-Chun Chen, Chung-I. Huang, Wei-Lun Huang, Yu-Wei Lin, Ching-Chieh Yang

**Affiliations:** 1 Department of Otolaryngology, Head and Neck Surgery, Kaohsiung Veterans General Hospital, Kaohsiung, Taiwan; 2 School of Medicine, National Defense Medical Center, Taipei, Taiwan; 3 Department of Otolaryngology, Head and Neck Surgery, Tri-Service General Hospital, Taipei, Taiwan; 4 Institute of Hospital and Health Care Administration, National Yang-Ming University, Taipei, Taiwan; 5 School of Medicine, National Yang-Ming University, Taipei, Taiwan; 6 Department of Hematology and Oncology, E-Da hospital, Kaohsiung, Taiwan; 7 School of Medicine, College of Medicine, I-Shou University, Kaohsiung, Taiwan; 8 Department of Radiation Oncology, Buddhist Dalin Tzu Chi Hospital, Chiayi, Taiwan; 9 School of Medicine, Tzu Chi University, Hualian, Taiwan; 10 Department of Radiation Oncology, Pingtung Christian Hospital, Pingtung, Taiwan; 11 Department of Radiation Oncology, E-Da Cancer Hospital, Kaohsiung, Taiwan; 12 Department of Radiation oncology, Kaohsiung Veterans General Hospital, Kaohsiung, Taiwan; 13 Department of Radiation Oncology, Chi-Mei Medical Center, Tainan, Taiwan; 14 Institute of Biomedical Sciences, National Sun Yat-Sen University, Kaohsiung, Taiwan; 15 Department of Pharmacy, Chia-Nan University of Pharmacy and Science, Tainan, Taiwan

## Abstract

To compare the prognostic value of 3 different lymph node scoring systems “ log odds of positive nodes (LODDS), lymph node ratio (rN), and lymph node yield “ in an effort to improve the staging of oral cancer. We identified 3958 oral cancer patients from Surveillance, Epidemiology, and End Results database from 2007 to 2013. In univariate analysis, LODDS, pN, rN, and lymph node yield were prognostic factors for 5-year disease-specific survival (DSS) and overall survival (OS). Multivariate analysis indicated that patients with LODDS 4 had worst 5-year DSS and OS. Stage migration occurred in pN1 and pN2 patients with LODDS 4. In pN1 patients, those with LODDS 4 had the worst 5-year DSS (41.2%) and OS (31.6%) than patients with pN1 and LODDS 2–3. In pN2 patients, those with LODDS4 had the worst 5-year DSS (34.5%) and OS (27.4%) than patients with pN2 and LODDS 2–3. The proposed staging system, which incorporates LODDS with AJCC pN, had better discriminability and prediction accuracy for predicting survival. We also noted that patients with LODDS 4 given adjuvant radiotherapy had better 5-year DSS and OS. The LODDS should be considered as a future candidate measurement for N category in oral cancer.

## Introduction

Most oncologists stage oral cancer using the American Joint Committee on Cancer (AJCC) tumor, node, metastasis (TNM) system, and also use this system for clinical decision-making and development of therapeutic strategies^1^. Although the most recent (8th edition) TNM classification for oral cancer, which considers extra-capsular extension, will be introduced in the near future, the impact of other clinical and pathological factors on survival indicates the need for a better staging tool^2,3^. One of the most important factors are lymph node number status, such as total lymph nodes retrieved, ratio of positive lymph nodes (rN), and log odds of positive lymph nodes (LODDS)^4–6^. Therefore, a study of the prognostic value of different lymph node scoring systems in oral cancer may aide in development of the forthcoming staging system.

Many studies have confirmed that the number of evaluated lymph nodes correlates with outcomes, and recommend that at least 18 lymph nodes be examined in patients with oral cancer^6,7^. Patients with inadequate lymph node harvests might experience stage migration and subsequent underestimation of disease severity^8,9^. Besides lymph node yield, other studies have analyzed the prognostic impact of rN–the ratio of positive lymph nodes to total examined nodes–in the staging of oral cancer^4,10^. In fact, the rN category is a reliable predictor of oral cancer outcomes^4^. Recent studies indicate that the LODDS may provide a more accurate prediction of survival than the AJCC pN and rN categories^11–13^. In particular, LODDS can discriminate among patients who have the same ratio of node metastasis but different survival rates, especially patients without positive lymph nodes or with an insufficient number of retrieved nodes^11,14^. Although there is limited literature on the use of LODDS in oral cancer, our prior studies showed that LODDS had better discriminability of oral cancer patients from different single institution^5,15^.

The objective of the current study of oral cancer patients was to use multivariate analysis to compare different measures of lymph node status–LODDS, AJCC pN, rN, and lymph node yield–to identify the system with the greatest prognostic power. We used the Surveillance, Epidemiology, and End Results (SEER) database to compare all features of patients, so that the large number of cases provides sufficient statistical power. We also propose a new staging system that can identify the high-risk group using an independent factor to adjust risk features for a future prospective study.

## Results

### Demographic data

Table 1 summarizes the demographic and clinical characteristics of the study cohort. There were 3958 cases with newly diagnosed oral cancer (2528 men [63.9%] and 1430 women [36.1%]). The mean age at diagnosis was 59 ± 13 years. About 52% of the patients had tongue cancer and 25% had inadequate lymph node dissection. The mean lymph node yield was 33 ± 17, and the mean number of positive lymph nodes was 1.31 ± 2.65.

**Table 1:** Demographic and clinical characteristics of the oral cancer patients, *n* = 3958.

Variables	Number, *n* (%)
Age (Mean ± SD)	59 ± 13
Gender	
Male	2528 (63.9%)
Female	1430 (36.1%)
Race	
White	3316 (83.8%)
Black/other	642 (16.2%)
Marital status	
Married	2240 (56.6%)
Other status	1718 (43.4%)
Tumor subsite	
Tongue	2041 (51.6%)
Lip	160 (4.0%)
Floor of mouth	671 (17.0%)
Gum and retromolar trigone	680 (17.2%)
Buccal mucosa	268 (6.8%)
Hard palate	55 (1.4%)
Other areas	83 (2.1%)
Differentiation	
Well/moderately	3060 (77.3%)
Poorly/undifferentiated	898 (22.7%)
Regional lymph nodes examined	
Adequate	2969 (75.0%)
Inadequate	989 (25.0%)
AJCC pT	
T1	1398 (35.3%)
T2	1353 (34.2%)
T3	474 (12.0%)
T4	733 (18.5%)
AJCC pN	
N0	2132 (53.9%)
N1	826 (20.9%)
N2	967 (24.4%)
N3	33 (0.8%)
LODDS	
LODDS1 (LODDS ≦ -1.68)	1360 (34.4%)
LODDS2 (-1.68 < LODDS ≦ -1.29)	1214 (30.7%)
LODDS3 (-1.29 < LODDS ≦ -0.88)	795 (20.1%)
LODDS4 (-0.88 < LODDS)	589 (14.9%)
rN	
N0 (rN = 0)	2139 (54.0%)
N1 (0 < rN ≦ 0.2)	1626 (41.1%)
N2 (0.2 < rN ≦ 0.4)	159 (4.0%)
N3 (0.4 < rN)	34 (0.9%)
Radiotherapy	
No	1851 (46.8%)
Yes	2107 (53.2%)
Year of diagnosis	
2007	432 (10.9%)
2008	488 (12.3%)
2009	588 (14.9%)
2010	575 (14.5%)
2011	587 (14.8%)
2012	648 (16.4%)
2013	640 (16.2%)

Abbreviation: LODDS, log odds of positive lymph nodes; rN, ratio-based lymph node system.

### Univariate and multivariate analysis of different lymph node measurements

**Figure 1:**
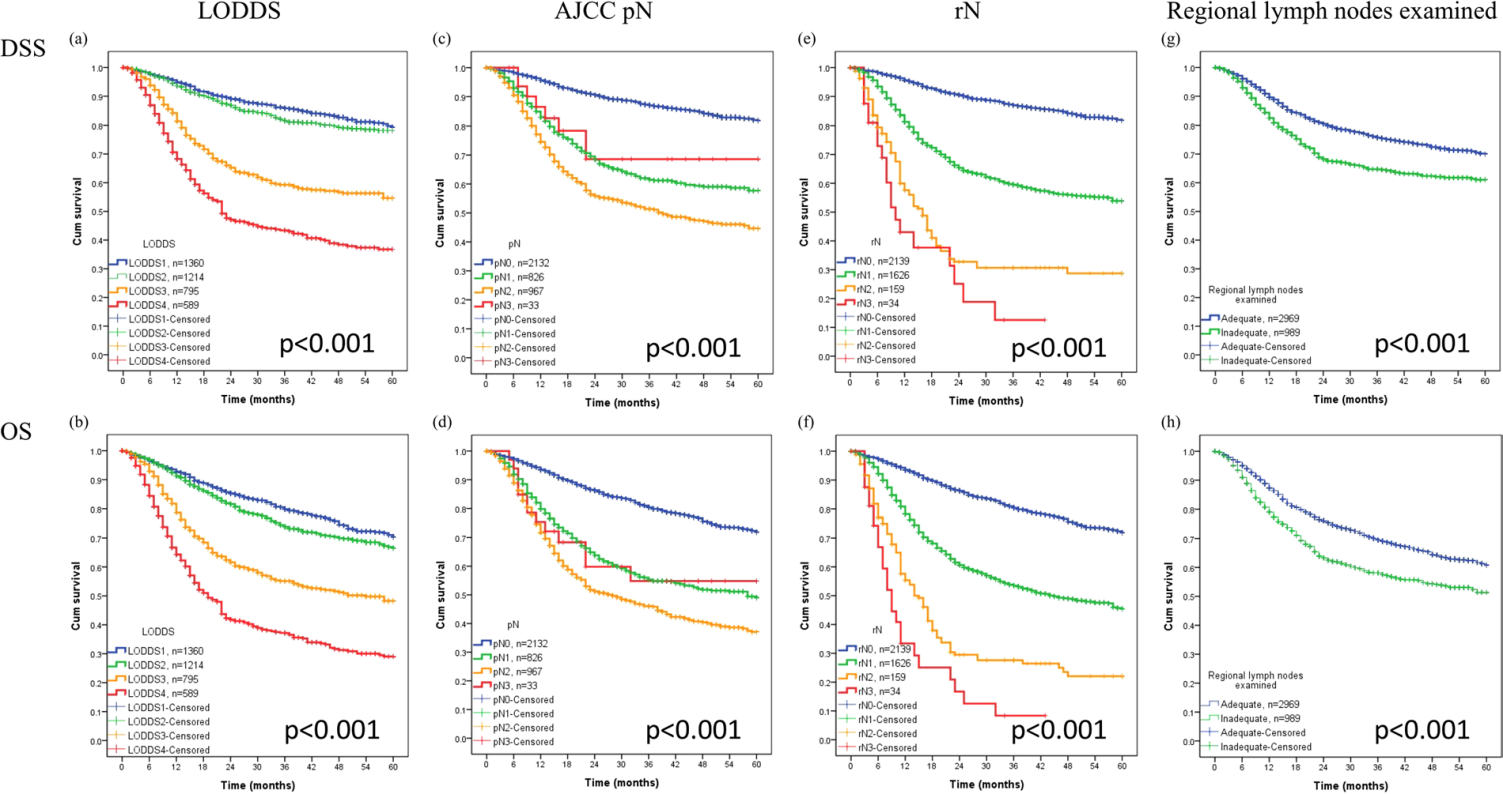
Kaplan-Meier curves for (**a**,**b**) LODDS in DSS and OS, (**c**,**d**) AJCC pN in DSS and OS, (**e**,**f**) rN in 5-year DSS and OS, and regional lymph nodes examined (**g**,**h**) from Surveillance, Epidemiology, and End Results (SEER) database.

Before our analysis, the multicollinearity and the reciprocal action effects among LODDS, AJCC pN, rN, and lymph node yield have been checked and presented no interaction between these measurements (Supplementary Table 1). Univariate analysis indicated that LODDS, pN, rN, and regional lymph nodes were significantly associated with 5-year DSS and OS (Fig. 1 and Supplementary Table 2). However, multivariate analysis indicated that only LODDS and rN measurements were significantly associated with 5-year OS (Table 2). In particular, patients with LODDS4 had poorer 5-year DSS (HR: 1.91, 95% CI: 1.28–2.83) and OS (HR: 1.86, 95% CI: 1.32–2.61) than those with LODDS1–3. Patients classified as rN3 had the worst 5-year OS (HR: 4.10, 95% CI, 0.93–18.19).

**Table 2:** Multivariate analysis for 5-year disease-specific survival and overall survival, *n* = 3958*.

Variables	Disease-specific survival			Overall survival		
	HR	95% CI	p value	HR	95% CI	p value
Age	1.02	(1.01–1.02)	<0.0001	1.02	(1.02–1.03)	<0.0001
Race						
White	1					
Black/other	1.11	(0.93–1.32)	0.250			
Marital status						
Married	1			1		
Other status	1.11	(0.97–1.28)	0.118	1.25	(1.11–1.41)	0.000
Tumor subsite						
Tongue	1			1		
Lip	0.43	(0.27–0.70)	0.001	0.57	(0.40–0.82)	0.002
Floor of mouth	1.08	(0.90–1.30)	0.423	1.14	(0.97–1.33)	0.123
Gum and retromolar trigone	0.94	(0.78–1.14)	0.551	0.98	(0.83–1.15)	0.765
Buccal mucosa	0.95	(0.73–1.24)	0.700	0.87	(0.68–1.11)	0.263
Hard palate	1.09	(0.67–1.79)	0.728	1.03	(0.65–1.62)	0.913
Other areas	1.16	(0.77–1.77)	0.480	1.25	(0.88–1.78)	0.215
Differentiation						
Well/moderately	1			1		
Poorly/undifferentiated	1.11	(0.96–1.29)	0.171	1.15	(1.01–1.31)	0.042
Regional lymph nodes examined						
Adequate	1			1		
Inadequate	0.89	(0.75–1.06)	0.187	0.93	(0.80–1.08)	0.317
AJCC pT						
T1	1			1		
T2	1.76	(1.45–2.14)	<0.0001	1.61	(1.37–1.90)	<0.0001
T3	3.04	(2.41–3.83)	<0.0001	2.79	(2.29–3.39)	<0.0001
T4	3.31	(2.67–4.10)	<0.0001	2.84	(2.36–3.42)	<0.0001
AJCC pN						
N0	1			1		
N1	1.83	(0.45–7.39)	0.397	1.20	(0.30–4.85)	0.796
N2	1.85	(0.46–7.49)	0.390	1.27	(0.31–5.11)	0.740
N3	0.94	(0.20–4.50)	0.941	0.87	(0.20–3.90)	0.858
LODDS						
LODDS1	1			1		
LODDS2	0.86	(0.66–1.12)	0.251	0.97	(0.79–1.20)	0.800
LODDS3	1.36	(0.95–1.94)	0.096	1.30	(0.96–1.77)	0.089
LODDS4	1.91	(1.28–2.83)	0.002	1.86	(1.32–2.61)	0.000
rN						
N0	1			1		
N1	1.36	(0.33–5.59)	0.668	1.72	(0.42–7.00)	0.450
N2	2.48	(0.59–10.42)	0.214	2.88	(0.69–11.97)	0.145
N3	4.10	(0.93–18.19)	0.063	5.25	(1.21–22.74)	0.027
Radiotherapy						
No	1			1		
Yes	0.78	(0.67–0.91)	0.002	0.71	(0.62–0.81)	<0.0001

Abbreviation: HR, hazard ratio; 95% CI, 95% confidence interval; LODDS, log odds of positive lymph nodes; rN, ratio-based lymph node system. *Variables with a *p* value < 0.05 in univairate analysis (Supplementary Table 2) were included in multivariate analysis.

### Stage migration in different pN categories

**Figure 2:**
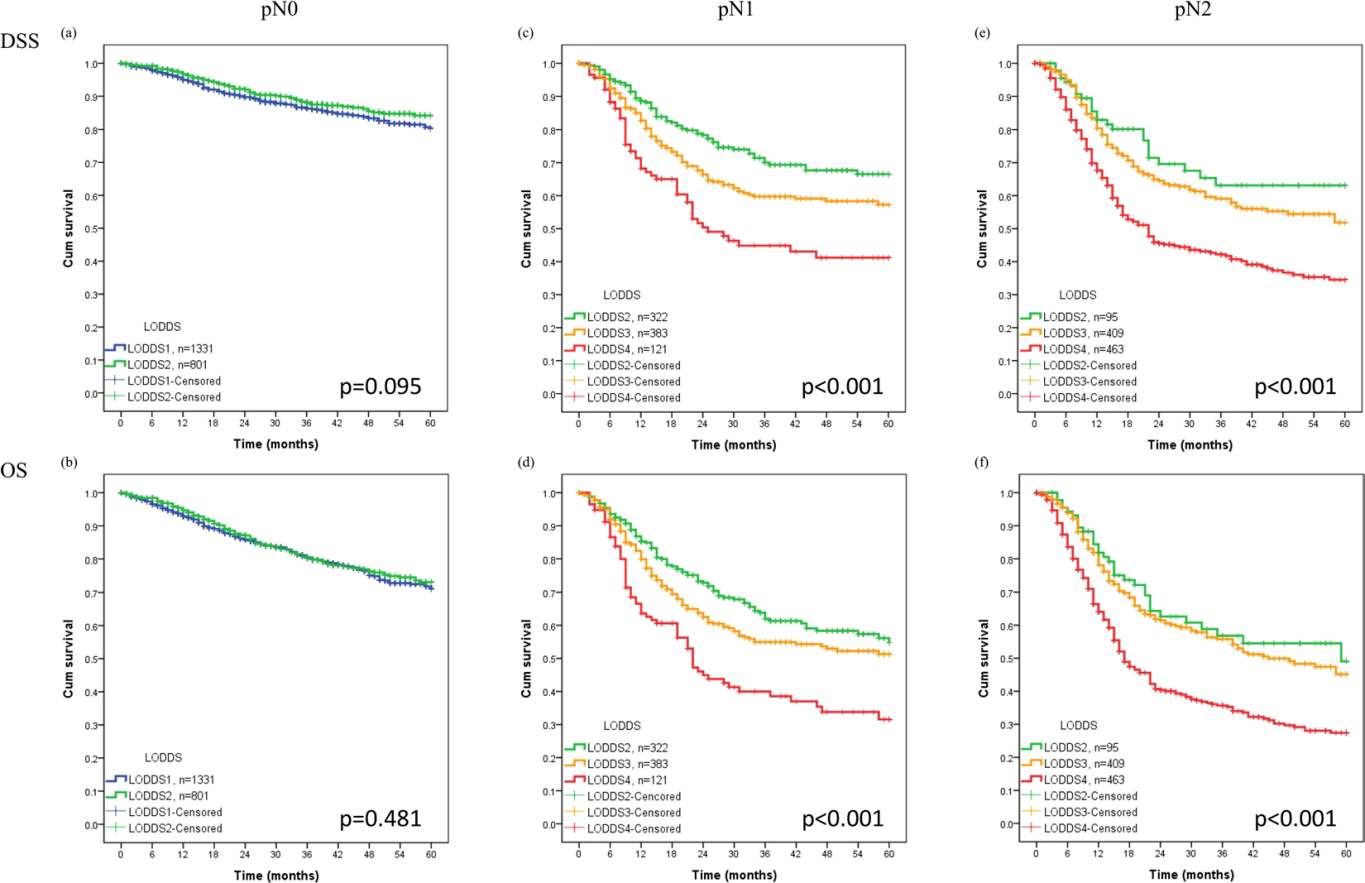
Kaplan-Meier curves for 5-year DSS and OS according to different AJCC pN plus LODDS.

We performed stratified analysis to check whether stage migration developed in the different pN and LODDS groups (Table 3 and Fig. 2). In pN0 patients, the LODDS1 and LODDS2 groups had similar survival rates; in pN1-2 patients, the LODDS4 group had the worst survival rate. Stage migration occurred in pN1 and pN2 patients with LODDS 4. In pN1 patients, those with LODDS4 had the worst 5-year DSS (41.2%) and OS (31.6%) than patients with pN1 and LODDS2-3. In pN2 patients, those with LODDS4 had the worst 5-year DSS (34.5%) and OS (27.4%) than patients with pN2 and LODDS2-3. We also examined the presence of stage migration for rN according to pN status, but observed no stage migration, presumably because only a few patients had rN3 disease (Supplementary Table 3).

**Table 3:** The 5-year overall survival and disease-specific survival of the oral cancer patients according to different AJCC pN plus LODDS, *n* = 3958.

New N category	Inclusion	AJCC pN	LODDS	Disease-specific survival			Overall survival		
				Case	Events	Survival rate (%)	Case	Events	Survival rate (%)
N0	✓	pN0	LODDS1	1331	159	80.3%	1331	239	71.1%
	✓	pN0	LODDS2	801	78	84.3%	801	137	73.1%
		pN0	LODDS3	0	0	—	0	0	—
		pN0	LODDS4	0	0	—	0	0	—
N1		pN1	LODDS1	23	9	33.8%	23	10	29.6%
	✓	pN1	LODDS2	314	72	66.5%	314	98	54.8%
	✓	pN1	LODDS3	375	119	57.3%	375	139	51.3%
N2	✓	pN1	LODDS4	114	54	41.2%	114	65	31.6%
		pN2	LODDS1	6	2	60.0%	6	2	60.0%
	✓	pN2	LODDS2	93	25	63.1%	93	33	49.1%
	✓	pN2	LODDS3	407	132	51.8%	407	152	45.1%
N3	✓	pN2	LODDS4	461	223	34.5%	461	263	27.4%
		pN3	LODDS1	0	0	—	0	0	—
		pN3	LODDS2	6	0	—	6	1	66.7%
		pN3	LODDS3	13	5	54.0%	13	7	44.9%
		pN3	LODDS4	14	3	71.8%	14	5	61.1%

Abbreviation: LODDS, log odds of positive lymph nodes.

### Performance of AJCC TNM stage and hypothetical system

**Figure 3:**
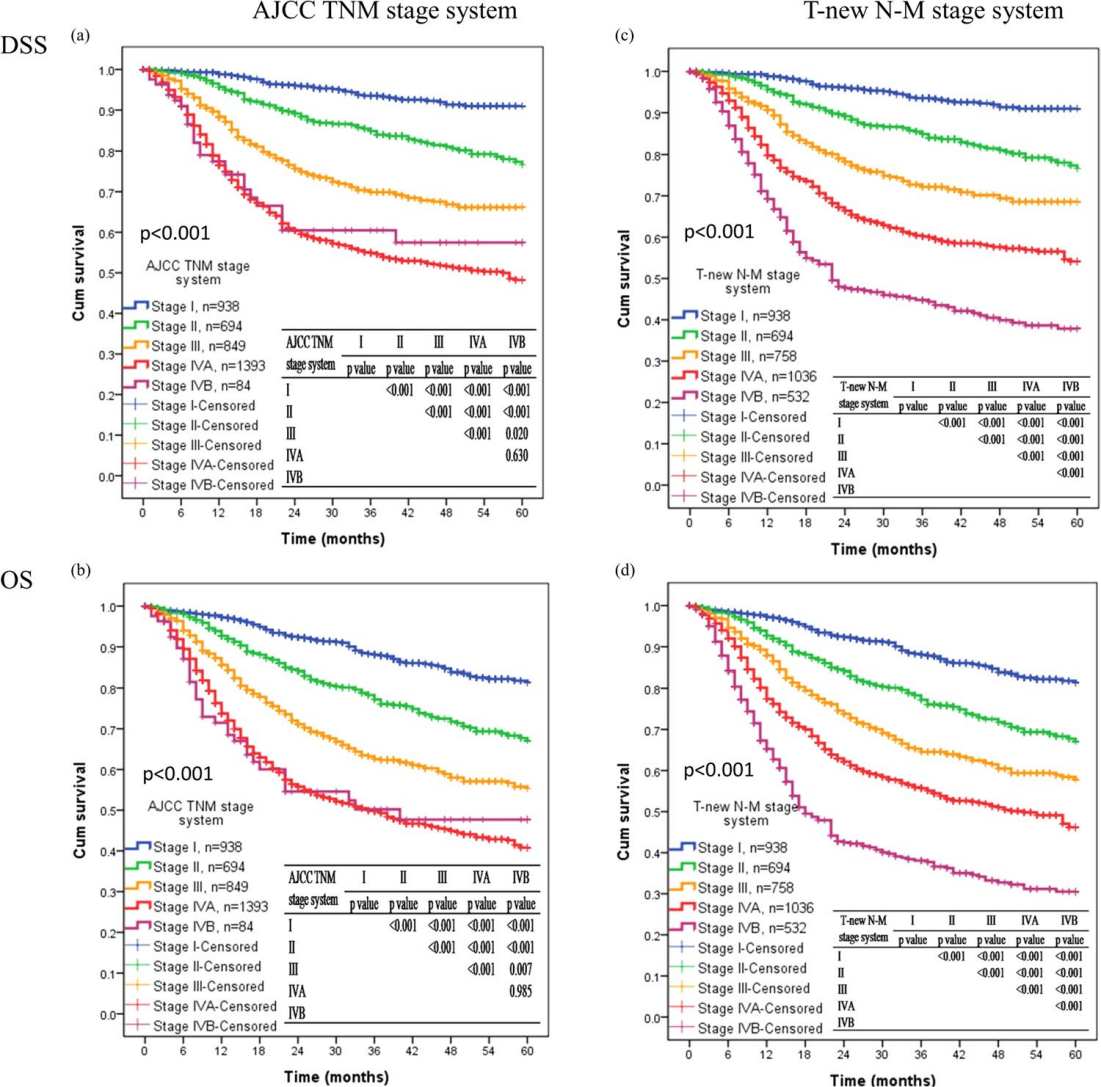
Kaplan-Meier curves for (**a**,**b**) TNM stage in DSS and OS, and (**c**,**d**) hypothetical T-new N-M system in 5-year DSS and OS.

Due to the effect of stage migration of pN1 and pN2 cancer, modification of N category was proposed, similar to our previous literature (Table 3)^15^. As described in the ‘Material and Methods’, subgroups with fewer than 30 OSCC patients were not included in this analysis. This led to a new N category in which each patient was placed into one of four groups: new N0 (pN0 and LODDS1-2); new N1 (pN1 and LODDS2-3); new N2 (pN1 and LODDS4, pN2 and LODDS2-3); and new N3 (pN2 and LODDS4). Thus, we compared the stage-specific DSS and OS survival rates according to current AJCC TNM staging and our new system (Fig. 3). There was no difference in DSS (*p* = 0.63) and OS (*p* = 0.985) between the AJCC stage IVa and IVb disease. However, the new system discriminated stage IVa and IVb in terms of DSS (*p* < 0.001) and OS (*p* < 0.001). In prediction of DSS, the new system outperformed the AJCC TNM system with a higher linear chi-square value (397 *vs*. 327), a lower AIC (13275 *vs*. 13329), and a higher Harrell’s C statistic (0.722 *vs*. 0.703) (Table 4). Similarly, a comparison of these 2 systems in terms of OS indicated the new system had higher linear chi-square (407 *vs*. 326), a lower AIC (17454 *vs*. 17514), and a higher Harrell’s C statistic (0.692 *vs*. 0.673). These results were in agreement with our previous single center study^15^. Moreover, our multivariate analysis indicated these results were robust (Table 5). Thus, the new model had better discriminatability and better prediction of DSS and OS than the existing AJCC TNM system. Supplementary Tables 4 and 5 show the detailed results of the Cox regression model.

**Table 4:** Discriminatory ability between AJCC TNM stage and T- New N-M stage system, *n* = 3958.

	Subgroups	Linear trend χ^2^	AIC	Harrell’s c-statistics
Performance of DSS prediction				
AJCC TNM stage	I, II, III, IVA, IVB	327	13329	0.703
T-New N-M stage	I, II, III, IVA, IVB	397	13275	0.722
Performance of OS prediction				
AJCC TNM stage	I, II, III, IVA, IVB	326	17514	0.673
T-New N-M stage	I, II, III, IVA, IVB	407	17454	0.692

Abbreviation: DSS, disease-specific survival; OS, overall survival; AIC, Akaike information criterion.

**Table 5:** Multivariate analysis of 5-year disease-specific survival & overall survival and model discrimination, *n* = 3958*.

Variables	Model 1: AJCC TNM-based model			Model 2: T-new N-M-based model		
	HR	95% CI	p value	HR	95% CI	p value
**DSS performance**						
AJCC pN						
N0	1					
N1	3.11	2.59–3.74	<0.001			
N2	4.38	3.66–5.24	<0.001			
N3	2.22	1.08–4.54	0.029			
New N category						
N0				1		
N1				2.86	2.35–3.47	<0.001
N2				3.53	2.89–4.30	<0.001
N3				5.79	4.75–7.06	<0.001
Discriminatory ability						
Linear trend χ^2^	273			316		
AIC	13164			13131		
Harrell’s c-statistic	0.757			0.762		
**OS performance**						
AJCC pN						
N0	1					
N1	2.55	2.18–2.98	< 0.001			
N2	3.55	3.05–4.14	< 0.001			
N3	2.49	1.42–4.38	0.001			
New N category						
N0				1		
N1				2.36	2.00–2.79	<0.001
N2				2.83	2.38–3.36	<0.001
N3				4.77	4.02–5.66	<0.001
Discriminatory ability						
Linear trend χ^2^	279			335		
AIC	17243			17200		
Harrell’s c-statistic	0.735			0.741		

*Adjusted for age, gender, tumor subsite, AJCC pT, differentiation, radiotherapy, marital status, race and year of diagnosis. Abbreviation: DSS, disease-specific survival; OS, overall survival; HR, hazard ratio; 95% CI, 95% confidence interval; AIC, Akaike information criterion.

### Role of adjuvant radiotherapy for high-risk patients

**Figure 4:**
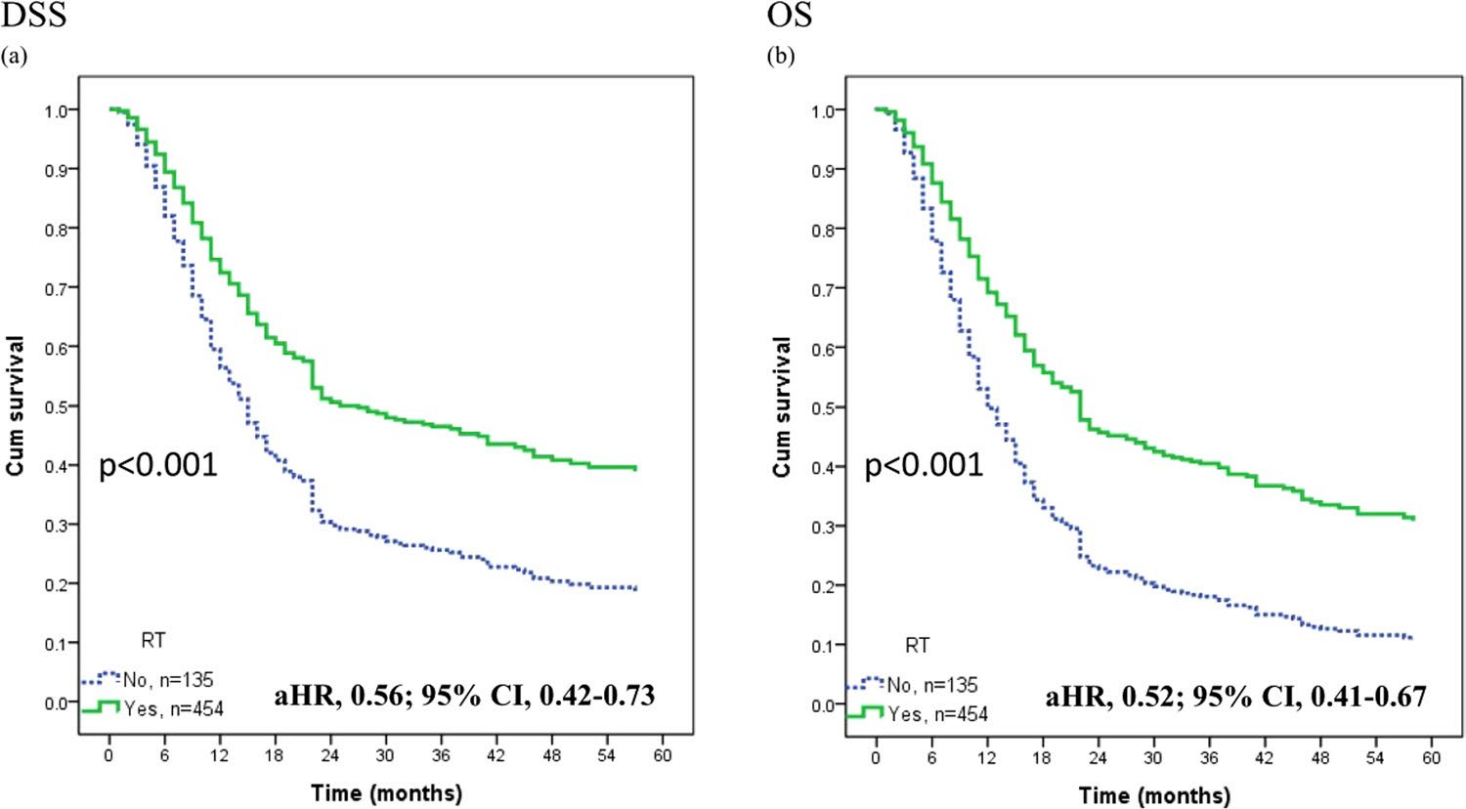
The adjusted Kaplan-Meier curves for adjuvant radiotherapy effect in 5-year DSS (**a**) and OS (**b**) among LODDS 4 patients.

We further analyzed the effect of adjuvant radiotherapy in patients with different LODDS. Among patients with LODDS 4, those treated with adjuvant radiotherapy had better 5-year DSS (aHR: 0.56, 95% CI: 0.42–0.73) and OS (aHR: 0.52; 95% CI: 0.41–0.67) than those not receiving adjuvant radiotherapy after adjusting other factors (Fig. 4).

## Discussion

We comprehensively analyzed the use of different lymph node scoring systems in patients with oral cancer using the SEER database, and validated the prognostic independency of LODDS for oral cancer in the United States. In particular, oral cancer patients with LODDS 4 (LODDS > -0.88) had 91% greater mortality in our 5-year DSS analysis and 86% greater mortality in our 5-year OS analysis relative to those with LODDS1 (LODDS ≤ -1.68) after adjustment for confounding. Stage migration occurred in pN1 and in pN2 with LODDS 4. The new system had better discriminability and prediction accuracy than the existing AJCC TNM system. Furthermore, our multivariate analysis indicated that high-risk patients (LODDS 4) benefitted from adjuvant radiotherapy. LODDS is therefore a reliable method that can be easily used by clinical practitioners, and should be considered as a future candidate measurement for nodal classification of oral cancer.

Currently, oral cancer patients with positive lymph nodes are staged as AJCC stage III–IV, and recommended for adjuvant radiotherapy or chemo-radiotherapy^16^. Due to multiple shortcomings of the N category in the current AJCC TNM staging system (7th edition), the new updated version (8^th^ edition) has incorporated extra-capsular spread in the clinical and pathological N category. However, there is continued discussion regarding the use of other prognostic factors, such as lymph node count, rN, and LODDS, for reducing stage migration^4,5,8^. This motivated our present research to assess the use of different lymph node scoring systems to develop a new N staging category that better stratifies high-risk patients for more intense therapy.

Multiple studies have investigated lymph node count as a prognostic factor in stomach cancer, colon cancer, and head and neck cancer, and also as a potential quality metric for neck dissection^7,8^. Divi *et al*. performed a large cohort study to examine these associations using a nation-wide database from the United States^8^. Their results showed an independent and significant association between fewer than 18 lymph nodes examined and increased risk of death (HR: 1.18, 95% CI: 1.13 to 1.22). In addition, when stratified by clinical nodal stage, there was an increased risk of death in the node-negative group (HR: 1.24, 95% CI: 1.17 to 1.32) and the node-positive group (HR: 1.12; 95% CI: 1.05 to 1.19). Thus, there is a significant overall survival advantage when more than 18 lymph nodes are examined after neck dissection. However, lymph node yield may depend on the specific hospitals, cancer severity, patient age, and patient performance status. Furthermore, the lymph node count may not reflect the real impact of positive lymph nodes on survival, so the lymph node ratio system should be still considered for pN stage assessment. The results of our univariate analysis showed a difference in DSS and OS according to LODDS, pN, rN, and regional lymph nodes examined (Fig. 1 and Supplementary Table 2). However, only LODDS and rN remained statistically significant in the multivariate analysis (Table 2). Thus, rN or LODDS system should be considered for improvement of the AJCC pN stage.

The current AJCC pN category for oral cancer is based on the size, number, and location of resected lymph nodes. However, when there is stage migration, the pN category underestimates the true extent of lymph node disease, and is therefore considered imperfect for prognostic purposes. For example, patients with the same pN classification, but a different number of examined nodes, will be given different prognoses. Therefore, the rN and LODDS systems, two new classifications for nodal estimation and behavior, are better than the traditional number-based pN system^9,17^. In our previous studies of oral cancer^5,15^, we found that LODDS performed better than the pN and rN systems. The main reason is that there is a non-linear association between the LODDS distribution and number of pathologically positive nodes^11^. Therefore, compared with rN, LODDS can discriminate among patients without positive lymph nodes or with a few positive nodes when insufficient nodes are retrieved. For example, a pN1 patient with a high LODDS should not be treated the same as a pN1 patient with low LODDS, because the former has a higher risk of occult metastases and worse prognosis. In this context, it is noteworthy that LODDS was a reliable lymph node measurement, and may be considered an alternative to pN stage.

Previous studies have successfully used LODDS in the study of breast, gastric, and colorectal cancer^11,17,18^. Besides our own former experience from two specific hospitals, there is little data on use of LODDS on outcome from oral cancer. Yildiz *et al*. reported a study of 225 surgically treated head and neck cancer patients, and reported LODDS as the only independent predictor for 5-year OS when comparing pN and rN^19^. The present SEER study confirms and validates that LODDS especially LOODS 4 is an independent prognostic factor, among various lymph node assessments, for oral cancer in the United States. In pN1 patients, those with LODDS4 had the worst 5-year DSS (41.2%) and OS (31.6%) than patients with pN1 and LODDS2-3. In pN2 patients, those with LODDS4 had the worst 5-year DSS (34.5%) and OS (27.4%) than patients with pN2 and LODDS2-3. Therefore LODDS 4 can compensate for the effect of migration of the AJCC pN stage. Furthermore, previous studies have outlined the importance of lymph node yield^7,8^. What is the value of LODDS in modification of oral cancer staging system? Our multivariate analysis indicated the lymph node yield was not an independent prognosticator when LODDS was in the model simultaneously. Furthermore, LODDS has better discriminability then lymph node yield and patients with inadequate node dissection had worse survival^8^. The current literature provides little advice for oral cancer patients who received inadequate node dissection. In the present study, LODDS helped to stratify patients and select the most appropriate therapy for those with LODDS4 (LODDS > -0.88, who had better prognosis when adjuvant radiotherapy was administered. In summary, lymph node yield could be regarded as “quality measure” of neck dissection, just as “surgical margin” may be a proxy of surgical technique. However, LODDS can be regarded as an reliable lymph node measurement for oral cancer, and an indicator of the suitability for adjuvant therapy.

There are several limitations in this study. First, the SEER database provides no information on whether the patients underwent bilateral neck dissection. We tried to minimize this confounding effect in lymph node yield, through exclusion of patients with pN2c disease, as previously suggested^6,20^. Moreover, we are currently conducting research using the Taiwan Cancer Database, which has clinical and pathological TNM data, and this may help to resolve this limitation. Second, the cutoff points for rN and LODDS were selected by our recent studies^5,21^. Modification of these cutoff points may be necessary to prevent subgroups with too few patients for analysis. Third, multivariable analysis indicated that lymph node yield was not an independent factor when LODDS was in the model. We did not perform further stratified analysis due to lack of statistical independence. Our study was not aimed to challenge the prognostic value of lymph node yield, but to find additional prognostic useful indicators. In fact, LODDS may be considered almost as a proxy of lymph node yield. Current guidelines recommend extensive extirpation of lymph nodes, without adverse damage to vessels and nerves, when performing neck dissection. Fourth, the SEER database provided no information on use of adjuvant chemotherapy, extra-capsular invasion and margin status. Some other unmeasured biases may also exist. Further research linked to Medicare claims (provide longitudinal utilization information for the cancer cases in SEER) or the use of instrumental variable analysis may help us to resolve these important issues^22^. Although this study describes the protective effect of adjuvant radiotherapy in those with LODDS4 after adjusting for confounding, these findings should be verified by a prospective study. Finally, the number of patients in some of our subgroups (pN0 with LODDS3-4; pN1 with LODDS1; pN2 with LODDS1; pN3) was small (fewer than 30) for survival analysis, so we did not estimate their survival rates using our new classification. Future researchers should consider recruitment of patients with pN3 disease, although several researchers recommend against surgical interventions for N3 disease due to the poor survival and high comorbidity of these patients^23^.

In conclusion, multivariate analysis indicated that LODDS4 was a reliable prognostic indicator for patients with oral cancer. We observed stage migration in patients classified as pN1 or as pN2 with LODDS4. This study validated that prognostic utility of the newly proposed system, which incorporated LODDS with AJCC pN, compared with AJCC TNM stage. The LODDS should be considered as a future reliable lymph node measurement for N category in oral cancer.

## Material and Methods

### Data source and study population

Data were obtained from the SEER database, sponsored by the National Cancer Institute, and consists of 18 population-based cancer registries. SEER data is an open access resource from United States used for cancer-based epidemiology and survival analyses. The Surveillance Research Program, using National Cancer Institute SEER*Stat software (seer.cancer.gov/seerstat) version 8.3.2, was used to identify eligible patients. All authors provided signed authorization to access this dataset. All methods were performed in accordance with the relevant guidelines and regulations of SEER database. The study design was approved by the Ethics Committee of the Institutional Review Board of Kaohsiung Veterans General Hospital.

Patients with new diagnoses of oral cancer after major surgery, with or without adjuvant radiotherapy, were identified from 2007 to 2013.Oral cancer patients were identified using the International Classification of Disease for Oncology, third edition (ICD-O-3). The ICD-O-3 categories included in this study were cancer of the lip (C00) and oral cavity (C02-C05.0; C06). To allow comparison of results with current AJCC N categories, any oral cancer patient without a clear AJCC TNM stage was excluded. All cases were staged according to the 6^th^ edition AJCC system^24^. Patients with a previous cancer, distant metastasis at initial diagnosis, pN2c disease, fewer than 10 examined LN, or who received any treatments prior to surgery (*e*.*g*. radiotherapy) were excluded. Patients with pN2c disease were also excluded because there may be confounding with the number of lymph nodes examined^20^. Patients with fewer than 10 lymph nodes examined may be regarded as not having received neck dissection according to our cancer center consensus. Finally, we examined the records of 3958 patients.

We examined the prognostic value of different features of neck lymph nodes in patients with oral cancer. The 3 lymph node scoring systems were:

1. Log odds of positive lymph node (LODDS).
  This is calculated as log_10_[(pnod + 0.5)/(tnod − pnod + 0.5)], in which pnod is the number of positive neck lymph nodes and tnod is the total number of cervical lymph nodes examined^25^. In this formula, 0.5 was added to the numerator and denominator to avoid division by 0. The cutoff points of LODDS were 35%, 60%, and 85% according to our previous publication^5^.
2. Number of cervical lymph nodes retrieved.
  This number was classified as adequate or inadequate, according to previous research^20^. Patients with pN0 disease with nodal yield more than 15 or those with pN1-3 disease with nodal yield more than 25 were categorized as having an adequate lymph nodes retrieval. All others were classified as having inadequate retrieval.
3. Ratio-based lymph node system (rN).

This ratio is calculated as the number of positive regional lymph nodes examined divided by the total number of regional lymph nodes examined. The cutoff points were 0.2 and 0.4, as in our previous study^21^.

### Measurements

The main endpoints were 5-year disease-specific survival (DSS) and overall survival (OS). Deaths from cancer and other conditions were extracted from the SEER database.

### Other variables

Basic characteristics, including age, sex, tumor subsite, AJCC pT, cell differentiation, receipt of radiotherapy, marital status, race, and year of diagnosis were also analyzed.

### Statistical analysis

All statistical analyses employed SPSS (version 15, SPSS Inc., Chicago, IL, USA). The 5-year OS and DSS rates for different lymph node scoring systems (LODDS, lymph node yield, pN, and rN,) were compared by the Kaplan-Meier method. Survival curves were measured from the time of initial diagnosis. Death from cancer was regarded as the event in DSS analysis, and death from all causes as the event in OS analysis. In multivariate analysis, the prognostic effect of different lymph node features were analyzed after adjusting for age, sex, T stage, cell differentiation, year of diagnosis, marital status, and treatment modality. The lymph node features that remained statistically significant during multivariate analyses were selected for further analysis. We also checked whether stage migration developed among patients in different AJCC N categories and with new lymph node features. Then, we constructed a new N staging system by adding LODDS into the AJCC pN category to improve the accuracy of 5 year predictions of DSS and OS, and compared the new staging system with the existing AJCC staging system. Subgroups with fewer than 30 OSCC patients were not included in the analysis because the small number of patients could lead to unreliable estimates of the 5-year DSS and OS.

Three indices were used to evaluate the prediction accuracy and discriminability of each model: a linear trend chi-square test, the Akaike information criterion (AIC), and Harrell’s C-statistic^14,26,27^. For Harrell’s C-statistic, a value of 0.5 indicates a value no better than chance; a value of 0.7–0.8 indicates an acceptable model; a value of 0.8–0.9 indicates an excellent model; and a value of 0.9–1 indicates an outstanding model. A linear trend chi-square test was used to assess monotonicity, in which a higher value indicates stronger monotonicity. Comparison of different staging system was also performed using mutltivariate analysis. A two-sided *p*-value below 0.05 was considered significant^28^.

## Electronic supplementary material

Supplementary Table 1–5
