# Supplementary material for: Recommendation for incorporation of a different lymph node scoring system in future AJCC N category for oral cancer
Source: Sci Rep. 2017 Oct 26;7:14117. doi: 10.1038/s41598-017-06452-0 (PMC5658398; doi:10.1038/s41598-017-06452-0)
Supplement: Supplementary file 1 — Supplementary Table 1–5 [file 41598_2017_6452_MOESM1_ESM.doc]

**Recommendation for incorporation of a different lymph node scoring system in future AJCC N category for oral cancer**

Ching-Chih Lee ^1,2,3^; Yu-Chieh Su ^4,5^; Shih-Kai Hung ^6,7^, Po-Chun Chen ^8^ ; Chung-I Huang ^9^; Wei-Lun Huang ^10^; Yu-Wei Lin ^11,12^; Ching-Chieh Yang ^11,12,13^*

^1^ Department of Otolaryngology, Head and Neck Surgery, Kaohsiung Veterans General Hospital, Kaohsiung, Taiwan

^2^ School of Medicine, National Defense Medical Center, Taipei, Taiwan

^3^ Department of Otolaryngology, Head and Neck Surgery, Tri-Service General Hospital, Taipei, Taiwan

^4^ Department of Hematology and Oncology, E-Da hospital, Kaohsiung, Taiwan

^5^ School of Medicine, College of Medicine, I-Shou University, Kaohsiung,Taiwan

^6^ Departments of Radiation Oncology, Buddhist Dalin Tzu Chi Hospital, Chiayi, Taiwan

^7^ School of Medicine, Tzu Chi University, Hualian, Taiwan

^8^ Department of Radiation Oncology, Pingtung Christian Hospital, Pingtung, Taiwan

^9^ Department of Radiation Oncology, E-Da Cancer Hospital, Kaohsiung, Taiwan

^10^ Department of Radiation oncology, Kaohsiung Veterans General Hospital, Kaohsiung, Taiwan.

^11^ Department of Radiation Oncology, Chi-Mei Medical Center, Tainan, Taiwan

^12^ Institute of Biomedical Sciences, National Sun Yat-Sen University, Kaohsiung, Taiwan

^13^ Department of Pharmacy, Chia-Nan University of Pharmacy and Science, Tainan, Taiwan

***Corresponding author:**

Ching-Chieh Yang

B2, No.901, Zhonghua Rd., Yongkang Dist., Tainan City 710, Taiwan (R.O.C.)

Telephone: +886 6 2812811 - 53501, Fax: +886 6 2820049

e-mail: [cleanclear0905@gmail.com](mailto:cleanclear0905@gmail.com)

**Running Title:**

LODDS added into pN improves survival prediction

| Supplementary table 1 |
| --- |
| Table 1-1. Pearson correlation between the predictors for disesase-specific survival (DSS)   |  | | DSS | LODDS | pN | rN | LN yield | | --- | --- | --- | --- | --- | --- | --- | | Pearson correlation | DSS | 1.000 | 0.300 | 0.287 | 0.282 | 0.030 | | LODDS | 0.300 | 1.000 | 0.789 | 0.848 | -0.338 | | pN | 0.287 | 0.789 | 1.000 | 0.628 | 0.118 | | rN | 0.282 | 0.848 | 0.628 | 1.000 | -0.118 | | LN yield | 0.030 | -0.338 | 0.118 | -0.118 | 1.000 |   ※ |
| Table 1-2. Detection of Multicollinearity based on collinearity statistics   | Model | | Unstandardized Coefficients | | p-value | Correlations | | | Collinearity Statistics | | | --- | --- | --- | --- | --- | --- | --- | --- | --- | --- | | Β | Std. Error | Zero-order | Partial | Part | Tolerance | VIF | | 1 | (Constant) | 0.579 | 0.076 | 0.000 |  |  |  |  |  | |  | LODDS | 0.147 | 0.023 | 0.000 | 0.300 | 0.101 | 0.096 | 0.063 | 15.932 | |  | pN | -0.016 | 0.018 | 0.366 | 0.287 | -0.014 | -0.014 | 0.159 | 6.300 | |  | rN | -0.014 | 0.165 | 0.932 | 0.282 | -0.001 | -0.001 | 0.189 | 5.303 | |  | LN yield | 0.004 | 0.001 | 0.000 | 0.030 | 0.100 | 0.095 | 0.336 | 2.978 |   a. Dependent Variable: DSS  Interpretations:  ①LODDS and LN yield were candidates for collinearity with p value<0.05 & VIF>2.5  ②We further added the interaction team in the correlation matrix |
| Table 1-3. Pearson correlation   |  | | DSS | LODDS | LN yield | LODDS×LN yield | | --- | --- | --- | --- | --- | --- | | Pearson correlation | DSS | 1.000 | 0.300 | 0.030 | 0.112 | | LODDS | 0.300 | 1.000 | -0.338 | 0.696 | | LN yield | 0.030 | -0.338 | 1.000 | -0.873 | | LODDS×LN yield | 0.112 | 0.696 | -0.873 | 1.000 |   ※ |

| Table 1-4. Detection of multicollinearity based on collinearity statistics   | Model | | Unstandardized Coefficients | | p-value | Correlations | | | Collinearity Statistics | | | --- | --- | --- | --- | --- | --- | --- | --- | --- | --- | | Β | Std. Error | Zero-order | Partial | Part | Tolerance | VIF | | 1 | (Constant) | 0.557 | 0.041 | 0.000 |  |  |  |  |  | |  | LODDS | 0.140 | 0.013 | 0.000 | 0.300 | 0.175 | 0.168 | 0.209 | 4.779 | |  | LN yield | 0.003 | 0.001 | 0.012 | 0.030 | 0.040 | 0.038 | 0.096 | 10.385 | |  | LODDS×LN yield | 0.000 | 0.000 | 0.558 | 0.112 | -0.009 | -0.009 | 0.056 | 17.890 |   a. Dependent Variable: DSS  Interpretations:  LODDS and LN yield were candidates for further analysis with a p-value<0.05 & VIF>2.5.  The interaction team was excluded (p-value=0.558). |
| --- | --- | --- | --- | --- | --- | --- | --- | --- | --- | --- | --- | --- | --- | --- | --- | --- | --- | --- | --- | --- | --- | --- | --- | --- | --- | --- | --- | --- | --- | --- | --- | --- | --- | --- | --- | --- | --- | --- | --- | --- | --- | --- | --- | --- | --- | --- | --- | --- | --- | --- | --- | --- | --- | --- | --- | --- | --- |
| Table 1-5. Detection of multicollinearity based on collinearity statistics   |  |  | Eigenvalue | Condition Index | Variance Proportions | | | | --- | --- | --- | --- | --- | --- | --- | | Model | Dimension | (Constant) | LODDS | LN yield | | 1 | 1 | 2.822 | 1.000 | 0.01 | 0.01 | 0.02 | |  | 2 | 0.127 | 4.722 | 0.13 | 0.11 | 0.98 | |  | 3 | 0.052 | 7.392 | 0.86 | 0.88 | 0.00 |   Interpretations:  ①There was no situation when both LODDS & LN yield had variance proportions>0.8.  ②There was no multicollinearity between LODDS & LN yield. |

| Table 1-6. Pearson correlation between the predictors for overall survival (OS)   |  | | DSS | LODDS | pN | rN | LN yield | | --- | --- | --- | --- | --- | --- | --- | | Pearson correlation | OS | 1.000 | 0.288 | 0.272 | 0.275 | 0.021 | | LODDS | 0.288 | 1.000 | 0.789 | 0.848 | -0.338 | | pN | 0.272 | 0.789 | 1.000 | 0.628 | 0.118 | | rN | 0.275 | 0.848 | 0.628 | 1.000 | -0.118 | | LN yield | 0.021 | -0.338 | 0.118 | -0.118 | 1.000 |   ※ |
| --- | --- | --- | --- | --- | --- | --- | --- | --- | --- | --- | --- | --- | --- | --- | --- | --- | --- | --- | --- | --- | --- | --- | --- | --- | --- | --- | --- | --- | --- | --- | --- | --- | --- | --- | --- | --- | --- | --- |
| Table 1-7. Detection of multicollinearity based on collinearity statistics   | Model | | Unstandardized Coefficients | | p-value | Correlations | | | Collinearity Statistics | | | --- | --- | --- | --- | --- | --- | --- | --- | --- | --- | | Β | Std. Error | Zero-order | Partial | Part | Tolerance | VIF | | 1 | (Constant) | 0.607 | 0.084 | 0.000 |  |  |  |  |  | |  | LODDS | 0.134 | 0.025 | 0.000 | 0.288 | 0.084 | 0.080 | 0.063 | 15.932 | |  | pN | -0.008 | 0.020 | 0.672 | 0.272 | -0.007 | -0.006 | 0.159 | 6.300 | |  | rN | 0.150 | 0.182 | 0.408 | 0.275 | 0.013 | 0.012 | 0.189 | 5.303 | |  | LN yield | 0.004 | 0.001 | 0.000 | 0.021 | 0.082 | 0.078 | 0.336 | 2.978 |  1. Dependent Variable: OS   Interpretations:  LODDS and LN yield were candidates for further analysis with a p-value<0.05 & VIF>2.5 |
| Table 1-8. Pearson correlation   |  | | OS | LODDS | LN yield | LODDS×LN yield | | --- | --- | --- | --- | --- | --- | | Pearson correlation | OS | 1.000 | 0.288 | 0.021 | 0.109 | | LODDS | 0.288 | 1.000 | -0.338 | 0.696 | | LN yield | 0.021 | -0.338 | 1.000 | -0.873 | | LODDS×LN yield | 0.109 | 0.696 | -0.873 | 1.000 |   ※ |

| Table 1-9. Detection of multicollinearity based on collinearity statistics   | Model | | Unstandardized Coefficients | | p-value | Correlations | | | Collinearity Statistics | | | --- | --- | --- | --- | --- | --- | --- | --- | --- | --- | | Β | Std. Error | Zero-order | Partial | Part | Tolerance | VIF | | 1 | (Constant) | 0.697 | 0.045 | 0.000 |  |  |  |  |  | |  | LODDS | 0.161 | 0.014 | 0.000 | 0.288 | 0.183 | 0.177 | 0.209 | 4.779 | |  | LN yield | 0.001 | 0.001 | 0.333 | 0.021 | 0.015 | 0.015 | 0.096 | 10.385 | |  | LODDS×LN yield | -0.001 | 0.000 | 0.062 | 0.109 | -0.030 | -0.028 | 0.056 | 17.830 |   a. Dependent Variable: DSS  Interpretations:  Only LODDS reached statistical significance.  No collinearity was noted, and no further collinearity analysis was necessary. |
| --- | --- | --- | --- | --- | --- | --- | --- | --- | --- | --- | --- | --- | --- | --- | --- | --- | --- | --- | --- | --- | --- | --- | --- | --- | --- | --- | --- | --- | --- | --- | --- | --- | --- | --- | --- | --- | --- | --- | --- | --- | --- | --- | --- | --- | --- | --- | --- | --- | --- | --- | --- | --- | --- | --- | --- | --- | --- |

Supplementary table 2. Univariate analysis for 5-year disease-specific survival and overall survival, *n*=3958

|  | Disease-specific survival | | |  | Overall survival | | |
| --- | --- | --- | --- | --- | --- | --- | --- |
| Variables | HR | 95% CI | p value |  | HR | 95% CI | p value |
| Age (Mean±SD) | 1.01 | 1.01-1.02 | <0.001 |  | 1.02 | 1.01-1.02 | <0.001 |
| Gender |  |  |  |  |  |  |  |
| Male | 1 |  |  |  | 1 |  |  |
| Female | 0.93 | 0.80-1.06 | 0.302 |  | 0.89 | 0.79-1.01 | 0.074 |
| Race |  |  |  |  |  |  |  |
| White | 1 |  |  |  | 1 |  |  |
| Black / other | 1.20 | 1.01-1.43 | 0.034 |  | 1.07 | 0.92-1.26 | 0.340 |
| Marital status |  |  |  |  |  |  |  |
| Married | 1 |  |  |  | 1 |  |  |
| Other status | 1.28 | 1.12-1.46 | <0.001 |  | 1.43 | 1.27-1.61 | <0.001 |
| Tumor subsite |  |  |  |  |  |  |  |
| Tongue | 1 |  |  |  | 1 |  |  |
| Lip | 0.56 | 0.35-0.90 | 0.017 |  | 0.78 | 0.54-1.11 | 0.178 |
| Floor of mouth | 1.22 | 1.01-1.46 | 0.032 |  | 1.31 | 1.12-1.53 | 0.001 |
| Gum and retromolar trigone | 1.37 | 1.15-1.63 | <0.001 |  | 1.42 | 1.22-1.66 | <0.001 |
| Buccal mucosa | 1.36 | 1.05-1.76 | 0.018 |  | 1.24 | 0.98-1.58 | 0.068 |
| Hard palate | 1.81 | 1.12-2.95 | 0.016 |  | 1.70 | 1.09-2.66 | 0.019 |
| Other areas | 1.79 | 1.19-2.68 | 0.004 |  | 1.97 | 1.40-2.78 | <0.001 |
| Differentiation |  |  |  |  |  |  |  |
| Well / moderately | 1 |  |  |  | 1 |  |  |
| Poorly / undifferentiated | 1.56 | 1.35-1.81 | <0.001 |  | 1.54 | 1.35-1.75 | <0.001 |
| Regional lymph nodes examined |  |  |  |  |  |  |  |
| Adequate | 1 |  |  |  | 1 |  |  |
| Inadequate | 1.54 | 1.34-1.78 | <0.001 |  | 1.48 | 1.31-1.68 | <0.001 |
| AJCC pT |  |  |  |  |  |  |  |
| T1 | 1 |  |  |  | 1 |  |  |
| T2 | 2.15 | 1.77-2.60 | <0.001 |  | 1.87 | 1.60-2.20 | <0.001 |
| T3 | 3.63 | 2.90-4.53 | <0.001 |  | 3.18 | 2.63-3.84 | <0.001 |
| T4 | 4.31 | 3.54-5.25 | <0.001 |  | 3.49 | 2.95-4.13 | <0.001 |
| AJCC pN |  |  |  |  |  |  |  |
| N0 | 1 |  |  |  | 1 |  |  |
| N1 | 3.22 | 2.69-3.84 | <0.001 |  | 2.50 | 2.15-2.91 | <0.001 |
| N2 | 4.78 | 4.06-5.62 | <0.001 |  | 3.59 | 3.13-4.12 | <0.001 |
| N3 | 2.48 | 1.22-5.03 | 0.011 |  | 2.56 | 1.47-4.46 | 0.001 |
| LODDS |  |  |  |  |  |  |  |
| LODDS1 | 1 |  |  |  | 1 |  |  |
| LODDS2 | 1.17 | 0.95-1.45 | 0.134 |  | 1.22 | 1.03-1.45 | 0.022 |
| LODDS3 | 3.12 | 2.57-3.80 | <0.001 |  | 2.48 | 2.10-2.94 | <0.001 |
| LODDS4 | 5.36 | 4.43-6.49 | <0.001 |  | 4.37 | 3.71-5.15 | <0.001 |
|  |  |  |  |  |  |  |  |
| rN |  |  |  |  |  |  |  |
| N0 | 1 |  |  |  | 1 |  |  |
| N1 | 3.54 | 3.04-4.13 | <0.001 |  | 2.75 | 2.42-3.12 | <0.001 |
| N2 | 8.58 | 6.74-10.93 | <0.001 |  | 6.18 | 4.96-7.69 | <0.001 |
| N3 | 13.90 | 8.79-21.98 | <0.001 |  | 11.35 | 7.56-17.05 | <0.001 |
| Radiotherapy |  |  |  |  |  |  |  |
| No | 1 |  |  |  | 1 |  |  |
| Yes | 1.77 | 1.54-2.04 | <0.001 |  | 1.42 | 1.27-1.60 | <0.001 |
| Year of diagnosis |  |  |  |  |  |  |  |
| 2007 | 1.01 | 0.65-1.57 | 0.940 |  | 0.99 | 0.68-1.45 | 0.979 |
| 2008 | 1.22 | 0.79-1.88 | 0.354 |  | 1.08 | 0.74-1.57 | 0.659 |
| 2009 | 1.06 | 0.69-1.62 | 0.786 |  | 1.01 | 0.70-1.47 | 0.931 |
| 2010 | 1.18 | 0.77-1.81 | 0.440 |  | 1.10 | 0.76-1.59 | 0.614 |
| 2011 | 1.23 | 0.80-1.90 | 0.326 |  | 1.18 | 0.81-1.71 | 0.382 |
| 2012 | 0.86 | 0.55-1.35 | 0.533 |  | 0.84 | 0.57-1.23 | 0.380 |
| 2013 | 1 |  |  |  | 1 |  |  |

Abbreviation: HR, hazard ratio; 95% CI, 95% confidence interval; LODDS, log odds of positive lymph nodes; rN, ratio-based lymph node system.

Supplementary table 3. The 5-year overall survival and disease-specific survival of the

oral cancer patients according to different AJCC pN plus rN, *n*=3958

|  | | Disease-specific survival | | |  | Overall survival | | |
| --- | --- | --- | --- | --- | --- | --- | --- | --- |
| AJCC pN | rN | Case | Events | Survival rate (%) |  | Case | Events | Survival rate (%) |
| pN0 | rN0 | 2132 | 237 | 81.8% |  | 2132 | 376 | 71.9% |
| pN0 | rN1 | 0 | 0 | -- |  | 0 | 0 | -- |
| pN0 | rN2 | 0 | 0 | -- |  | 0 | 0 | -- |
| pN0 | rN3 | 0 | 0 | -- |  | 0 | 0 | -- |
| pN1 | rN0 | 4 | 0 | -- |  | 4 | 0 | -- |
| pN1 | rN1 | 796 | 236 | 58.6% |  | 796 | 291 | 49.9% |
| pN1 | rN2 | 23 | 15 | 29.0% |  | 23 | 18 | 18.7% |
| pN1 | rN3 | 3 | 3 | 0.0% |  | 3 | 3 | 0.0% |
| pN2 | rN0 | 3 | 2 | 33.3% |  | 3 | 2 | 33.3% |
| pN2 | rN1 | 802 | 288 | 48.5% |  | 802 | 343 | 40.6% |
| pN2 | rN2 | 132 | 76 | 26.8% |  | 132 | 84 | 21.1% |
| pN2 | rN3 | 30 | 16 | 16.0% |  | 30 | 21 | 10.0% |
| pN3 | rN0 | 0 | 0 | -- |  | 0 | 0 | -- |
| pN3 | rN1 | 28 | 6 | 71.0% |  | 28 | 11 | 54.4% |
| pN3 | rN2 | 4 | 1 | 75.0% |  | 4 | 1 | 75.0% |
| pN3 | rN3 | 1 | 1 | 0.0% |  | 1 | 1 | 0.0% |

Abbreviation: LODDS, log odds of positive lymph nodes.

Supplementary table 4. Multivariate analysis of disease-specific survival and model discrimination, *n*=3958*

|  | Model 1: AJCC TNM-based model | | |  | Model 2: T-new N-M-based model | | |
| --- | --- | --- | --- | --- | --- | --- | --- |
| Variables | HR | 95% CI | p value |  | HR | 95% CI | p value |
| Age (Mean±SD) | 1.01 | 1.01-1.02 | <0.001 |  | 1.01 | 1.01-1.02 | <0.001 |
| Gender |  |  |  |  |  |  |  |
| Male | 1 |  |  |  | 1 |  |  |
| Female | 0.92 | 0.80-1.06 | 0.295 |  | 0.91 | 0.79-1.05 | 0.236 |
| Tumor subsite |  |  |  |  |  |  |  |
| Tongue | 1 |  |  |  | 1 |  |  |
| Lip | 0.44 | 0.27-0.71 | 0.001 |  | 0.42 | 0.26-0.68 | <0.001 |
| Floor of mouth | 1.00 | 0.83-1.21 | 0.927 |  | 1.02 | 0.85-1.23 | 0.808 |
| Gum and retromolar trigone | 0.92 | 0.76-1.11 | 0.408 |  | 0.92 | 0.76-1.11 | 0.426 |
| Buccal mucosa | 0.99 | 0.76-1.29 | 0.953 |  | 0.98 | 0.75-1.27 | 0.878 |
| Hard palate | 1.04 | 0.63-1.71 | 0.871 |  | 1.02 | 0.62-1.68 | 0.924 |
| Other areas | 1.04 | 0.68-1.58 | 0.848 |  | 1.01 | 0.67-1.54 | 0.933 |
| AJCC pT |  |  |  |  |  |  |  |
| T1 | 1 |  |  |  | 1 |  |  |
| T2 | 1.76 | 1.45-2.14 | <0.001 |  | 1.74 | 1.43-2.12 | <0.001 |
| T3 | 2.82 | 2.24-3.55 | <0.001 |  | 2.83 | 2.25-3.57 | <0.001 |
| T4 | 3.22 | 2.60-3.99 | <0.001 |  | 3.24 | 2.62-4.02 | <0.001 |
| AJCC pN |  |  |  |  |  |  |  |
| N0 | 1 |  |  |  |  |  |  |
| N1 | 3.11 | 2.59-3.74 | <0.001 |  |  |  |  |
| N2 | 4.38 | 3.66-5.24 | <0.001 |  |  |  |  |
| N3 | 2.22 | 1.08-4.54 | 0.029 |  |  |  |  |
| New N category |  |  |  |  |  |  |  |
| N0 |  |  |  |  | 1 |  |  |
| N1 |  |  |  |  | 2.86 | 2.35-3.47 | <0.001 |
| N2 |  |  |  |  | 3.53 | 2.89-4.30 | <0.001 |
| N3 |  |  |  |  | 5.79 | 4.75-7.06 | <0.001 |
| Differentiation |  |  |  |  |  |  |  |
| Well / moderately | 1 |  |  |  | 1 |  |  |
| Poorly / undifferentiated | 1.16 | 1.00-1.35 | 0.043 |  | 1.13 | 0.97-1.31 | 0.098 |
| Radiotherapy |  |  |  |  |  |  |  |
| No | 1 |  |  |  | 1 |  |  |
| Yes | 0.79 | 0.68-0.93 | 0.004 |  | 0.77 | 0.66-0.90 | 0.001 |
| Marital status |  |  |  |  |  |  |  |
| Married | 1 |  |  |  | 1 |  |  |
| Other status | 1.16 | 1.01-1.32 | 0.032 |  | 1.16 | 1.01-1.32 | 0.032 |
| Race |  |  |  |  |  |  |  |
| White | 1 |  |  |  | 1 |  |  |
| Black / other | 1.10 | 0.92-1.31 | 0.266 |  | 1.12 | 0.94-1.33 | 0.188 |
|  |  |  |  |  |  |  |  |
| Year of diagnosis |  |  |  |  |  |  |  |
| 2007 | 1.09 | 0.70-1.69 | 0.689 |  | 1.08 | 0.69-1.67 | 0.724 |
| 2008 | 1.30 | 0.84-2.00 | 0.225 |  | 1.29 | 0.84-1.98 | 0.245 |
| 2009 | 1.11 | 0.72-1.71 | 0.611 |  | 1.14 | 0.74-1.76 | 0.532 |
| 2010 | 1.27 | 0.83-1.95 | 0.265 |  | 1.26 | 0.82-1.93 | 0.287 |
| 2011 | 1.23 | 0.80-1.89 | 0.337 |  | 1.26 | 0.82-1.93 | 0.289 |
| 2012 | 0.92 | 0.59-1.44 | 0.724 |  | 0.93 | 0.59-1.45 | 0.763 |
| 2013 | 1 |  |  |  | 1 |  |  |
| Discriminatory ability |  | | |  |  | | |
| Linear trend χ^2^ | 273 | | |  | 316 | | |
| Akaike information criterion | 13164 | | |  | 13131 | | |
| Harrell’s c-statistic | 0.757 | | |  | 0.762 | | |

*Adjusted for age, gender, tumor subsite, AJCC pT, differentiation, radiotherapy, marital status, race and year of diagnosis.

Abbreviation: HR, hazard ratio; 95% CI, 95% confidence interval; AIC, Akaike information criterion.

Supplementary table 5. Multivariate analysis of overall survival and model discrimination, *n*=3958*

|  | Model 1: AJCC TNM-based model | | |  | Model 2: T-new N-M-based model | | |
| --- | --- | --- | --- | --- | --- | --- | --- |
| Variables | HR | 95% CI | p value |  | HR | 95% CI | p value |
| Age (Mean±SD) | 1.02 | 1.01-1.02 | <0.001 |  | 1.02 | 1.01-1.02 | <0.001 |
| Gender |  |  |  |  |  |  |  |
| Male | 1 |  |  |  | 1 |  |  |
| Female | 0.85 | 0.75-0.96 | 0.013 |  | 0.84 | 0.74-0.95 | 0.008 |
| Tumor subsite |  |  |  |  |  |  |  |
| Tongue | 1 |  |  |  | 1 |  |  |
| Lip | 0.56 | 0.39-0.81 | 0.002 |  | 0.55 | 0.38-0.80 | 0.002 |
| Floor of mouth | 1.07 | 0.91-1.26 | 0.386 |  | 1.09 | 0.93-1.28 | 0.280 |
| Gum and retromolar trigone | 0.96 | 0.81-1.13 | 0.629 |  | 0.96 | 0.81-1.13 | 0.643 |
| Buccal mucosa | 0.91 | 0.71-1.16 | 0.480 |  | 0.90 | 0.70-1.14 | 0.396 |
| Hard palate | 1.01 | 0.64-1.60 | 0.949 |  | 0.98 | 0.62-1.56 | 0.963 |
| Other areas | 1.14 | 0.80-1.62 | 0.461 |  | 1.14 | 0.80-1.63 | 0.453 |
| AJCC pT |  |  |  |  |  |  |  |
| T1 | 1 |  |  |  | 1 |  |  |
| T2 | 1.62 | 1.37-1.91 | <0.001 |  | 1.60 | 1.36-1.89 | <0.001 |
| T3 | 2.59 | 2.13-3.16 | <0.001 |  | 2.60 | 2.14-3.17 | <0.001 |
| T4 | 2.75 | 2.28-3.30 | <0.001 |  | 2.76 | 2.30-3.32 | <0.001 |
| AJCC pN |  |  |  |  |  |  |  |
| N0 | 1 |  |  |  |  |  |  |
| N1 | 2.55 | 2.18-2.98 | <0.001 |  |  |  |  |
| N2 | 3.55 | 3.05-4.14 | <0.001 |  |  |  |  |
| N3 | 2.49 | 1.42-4.38 | 0.001 |  |  |  |  |
| New N category |  |  |  |  |  |  |  |
| N0 |  |  |  |  | 1 |  |  |
| N1 |  |  |  |  | 2.36 | 2.00-2.79 | <0.001 |
| N2 |  |  |  |  | 2.83 | 2.38-3.36 | <0.001 |
| N3 |  |  |  |  | 4.77 | 4.02-5.66 | <0.001 |
| Differentiation |  |  |  |  |  |  |  |
| Well / moderately | 1 |  |  |  | 1 |  |  |
| Poorly / undifferentiated | 1.19 | 1.04-1.35 | 0.009 |  | 1.16 | 1.02-1.32 | 0.023 |
| Radiotherapy |  |  |  |  |  |  |  |
| No | 1 |  |  |  | 1 |  |  |
| Yes | 0.71 | 0.62-0.81 | <0.001 |  | 0.69 | 0.61-0.79 | <0.001 |
| Marital status |  |  |  |  |  |  |  |
| Married | 1 |  |  |  | 1 |  |  |
| Other status | 1.30 | 1.15-1.46 | <0.001 |  | 1.30 | 1.15-1.46 | <0.001 |
| Race |  |  |  |  |  |  |  |
| White | 1 |  |  |  | 1 |  |  |
| Black / other | 1.02 | 0.87-1.19 | 0.776 |  | 1.04 | 0.88-1.21 | 0.631 |
|  |  |  |  |  |  |  |  |
| Year of diagnosis |  |  |  |  |  |  |  |
| 2007 | 1.04 | 0.71-1.52 | 0.814 |  | 1.02 | 0.70-1.50 | 0.881 |
| 2008 | 1.14 | 0.78-1.66 | 0.483 |  | 1.12 | 0.77-1.63 | 0.533 |
| 2009 | 1.05 | 0.72-1.52 | 0.791 |  | 1.07 | 0.74-1.55 | 0.702 |
| 2010 | 1.15 | 0.79-1.67 | 0.441 |  | 1.14 | 0.79-1.66 | 0.467 |
| 2011 | 1.15 | 0.79-1.67 | 0.450 |  | 1.17 | 0.81-1.70 | 0.396 |
| 2012 | 0.88 | 0.60-1.30 | 0.541 |  | 0.89 | 0.60-1.32 | 0.586 |
| 2013 | 1 |  |  |  | 1 |  |  |
| Discriminatory ability |  | | |  |  | | |
| Linear trend χ^2^ | 279 | | |  | 335 | | |
| Akaike information criterion | 17243 | | |  | 17200 | | |
| Harrell’s c-statistic | 0.735 | | |  | 0.741 | | |

*Adjusted for age, gender, tumor subsite, AJCC pT, differentiation, radiotherapy, marital status, race and year of diagnosis.

Abbreviation: HR, hazard ratio; 95% CI, 95% confidence interval; AIC, Akaike information criterion.
